# The COORDINATE Pilot Study: Impact of a Transcatheter Aortic Valve Coordinator Program on Hospital and Patient Outcomes

**DOI:** 10.3390/jcm11051205

**Published:** 2022-02-23

**Authors:** Katja Bohmann, Christof Burgdorf, Tobias Zeus, Michael Joner, Héctor Alvarez, Kira Lisanne Berning, Maren Schikowski, Albert Markus Kasel, Gesine van Mark, Cornelia Deutsch, Jana Kurucova, Martin Thoenes, Derk Frank, Steffen Wundram, Peter Bramlage, Barbara Miller, Verena Veulemans

**Affiliations:** 1 Cardiothoracic Surgery Department, Heart and Vessel Center Bad Bevensen, 29549 Bad Bevensen, Germany; k.bohmann@hgz-bb.de (K.B.); m.schikowski@hgz-bb.de (M.S.); 2 Cardiology Department, Heart and Vessel Center Bad Bevensen, 29549 Bad Bevensen, Germany; c.burgdorf@hgz-bb.de; 3 Division of Cardiology, Pulmonology and Vascular Medicine, Medical Faculty, Heinrich Heine University, 40225 Düsseldorf, Germany; zeus@med.uni-duesseldorf.de (T.Z.); kiralisanne.berning@med.uni-duesseldorf.de (K.L.B.); verena.veulemans@med.uni-duesseldorf.de (V.V.); 4 German Heart Center Munich, 80636 Munich, Germany; joner@dhm.mhn.de (M.J.); alvarez_hector23@hotmail.com (H.A.); markus.kasel@usz.ch (A.M.K.); 5 Cardiology Department, University Heart Centre, University Hospital Zurich, 8091 Zurich, Switzerland; 6 Institute for Pharmacology and Preventive Medicine, 49661 Cloppenburg, Germany; gesine.van.mark@ippmed.de (G.v.M.); cornelia.deutsch@ippmed.de (C.D.); peter.bramlage@ippmed.de (P.B.); 7 Edwards Lifesciences, 18600 Prague, Czech Republic; jana_kurucova@edwards.com; 8 Edwards Lifesciences, 1260 Nyon, Switzerland; martin_thoenes@edwards.com; 9 Internal Medicine III (Cardiology, Angiology and Intensive Care Medicine) Department, UKSH University Clinical Center Schleswig-Holstein, 24105 Kiel, Germany; steffen.wundram@uksh.de; 10 German Centre for Cardiovascular Research, Partner Site Hamburg/Kiel/Lübeck, 24105 Kiel, Germany; 11 Cmillers, 97082 Würzburg, Germany; b.miller@cmillers.de

**Keywords:** TAVI, transcatheter aortic valve implantation, coordinator, patient pathways, patient safety

## Abstract

The transcatheter aortic valve implantation (TAVI) treatment pathway is complex, leading to procedure-related delays. Dedicated TAVI coordinators can improve pathway efficiency. COORDINATE was a pilot observational prospective registry at three German centers that enrolled consecutive elective patients with severe aortic stenosis undergoing TAVI to investigate the impact a TAVI coordinator program. Pathway parameters and clinical outcomes were assessed before (control group) and after TAVI coordinator program implementation (intervention phase). The number of repeated diagnostics remained unchanged after implementation. Patients with separate hospitalizations for screening and TAVI had long delays, which increased after implementation (65 days pre- vs. 103 days post-implementation); hospitalizations combining these were more efficient. The mean time between TAVI and hospital discharge remained constant. Nurse (*p* = 0.001) and medical technician (*p* = 0.008) working hours decreased. Patient satisfaction increased, and more consistent/intensive contact between patients and staff was reported. TAVI coordinators provided more post-TAVI support, including discharge management. No adverse effects on post-procedure or 30-day outcomes were seen. This pilot suggests that TAVI coordinator programs may improve aspects of the TAVI pathway, including post-TAVI care and patient satisfaction, without compromising safety. These findings will be further investigated in the BENCHMARK registry.

## 1. Introduction

The standard treatment for severe symptomatic aortic stenosis (AS) is transcatheter aortic valve implantation (TAVI) [1,2,3]. TAVI is safe and effective, but the treatment pathway is complex and can be affected by delays, patient complexities (i.e., increased age, comorbidities, and progressive disease) and multidisciplinary complexities that impact the quality of patient management and hospital efficiency [4,5,6]. In addition, reimbursement in Germany requires comprehensive documentation and correct coding. 

A recent concept is to have a dedicated TAVI coordinator, whose role is to support both patients and hospital staff alike, achieve an efficient treatment pathway, avoid duplicate diagnostic procedures, and facilitate communication and effective decision-making processes [4,7]. Several local initiatives have provided evidence for the benefits of a dedicated TAVI coordinator [4,7,8,9], but there has been no prospective documentation of the benefits across multiple institutions. The COORDINATE registry is a pilot study to prospectively document the impact of introducing a TAVI coordinator program at three different German hospitals. We hypothesized that establishing and training a dedicated TAVI coordinator would make the flow of patients more efficient without compromising patient safety. The findings from this pilot study will be further investigated and validated in the BENCHMARK registry (ClinicalTrials.gov Identifier: NCT04579445).

## 2. Materials and Methods

COORDINATE was a pilot observational prospective multicenter registry of patients undergoing balloon-expandable valve implantation at three different German institutions: the German Heart Center Munich; the Cardiology Department, Düsseldorf University; and the Heart Center Bad Bevensen. This investigation conforms to the principles outlined in the Declaration of Helsinki and complied with local laws and regulations. The ethics committee responsible for each site granted approval, and written informed consent was obtained from patients.

### 2.1. Patient Selection

Patients comprised consecutive elective patients with a diagnosis of severe symptomatic AS who were admitted for and underwent expandable valve implantation at one of the participating centers. To enable the broadest possible patient spectrum, and the full patient pathway from the diagnosis of severe AS to the post-TAVI implantation follow-up, no other inclusion or exclusion criteria were applied.

### 2.2. COORDINATE Study Phases and Procedures

**Figure 1:**
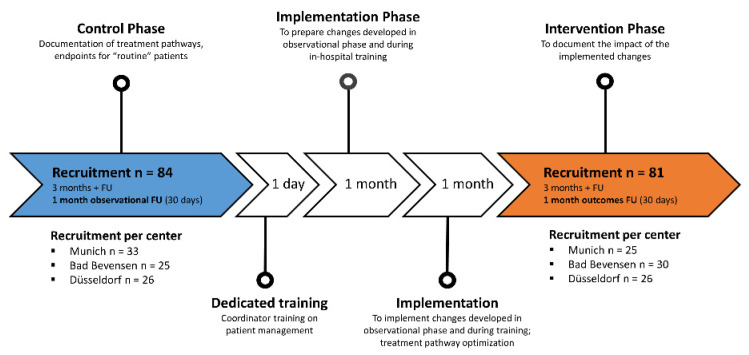
Study flow chart. FU, follow-up.

The registry comprised three study phases: (1) a control phase, (2) a training and “phase-in” enrollment (implementation phase), and (3) an intervention phase (Figure 1).

The control phase comprised a recruitment period (a maximum of 3 months to recruit a target of 25 patients/center) and a 1-month intervention observational follow-up period. The role of the TAVI coordinator during this phase was to document the existing (unaltered) clinical routine in an electronic case report form (eCRF) and attend heart team meetings to record the decision-making process. Further responsibilities included documentation of baseline patient characteristics, diagnostic procedures, referring physician communications, procedure-related variables, and details of the discharge process. 

The implementation phase started with a 1-day training session. Here, the three German centers convened for a critical appraisal of the existing treatment pathways, received training from an experienced site in Kiel with an established TAVI coordinator program, and developed center-specific action plans and standardized tools (e.g., checklists for TAVI coordinators and patient explanation sheets). A 1-month “phase-in” period followed, during which the changes were developed and agreed upon at each center. Finally, centers were allowed 1 month to implement their specific changes in preparation for patient enrollment into the TAVI coordinator program.

The intervention phase comprised a recruitment period (a maximum of 3 months to recruit a target of 25 patients/center) and a 1-month follow-up/patient. During this phase, the effects of the training, phase-in, and implementation of the TAVI coordinator program on hospital outcomes (e.g., duplicate pre-procedural assessments, time from hospital admission to the TAVI procedure, and time from TAVI to hospital discharge) were documented. 

Data were captured in an eCRF provided by Software for Trials Europe GmbH (Berlin, Germany), including data from hospital admission, diagnostic workup, heart team discussion, TAVI procedure, and hospital discharge. Diagnostic procedures being performed either prior to admission at an external site, or within the hospital performing the TAVI procedure, were documented. Patient follow-up was for 30 ± 7 days.

### 2.3. Coordinator Skill Set

Each center had a single TAVI coordinator: either a nurse or medical technical assistant (with the exception of the German Heart Center in Munich, which had a guest physician from South America), who was previously employed at or newly recruited to the center specifically for the role. The TAVI coordinator role was part-time (~20 h per week) and initially based on observation and documentation; full training was subsequently provided. 

### 2.4. Coordinator Responsibilities

A list of potential TAVI coordinator requirements is presented in Table 1, which was the result of a brainstorming session among the three centers to determine the most obvious, important, and realistic requirements (e.g., streamlining the diagnostic work-up and planning early discharge). Some centers had already optimized some of these requirements (illustrated with an empty box in Table 1), while other areas had the potential for improvement. As a result, there was no uniform baseline across all three centers. It was agreed, therefore, that improvements over baseline were the intervention/the potential for improvement. Each center chose 7–8 responsibilities to implement according to their center-specific requirements.

### 2.5. Outcomes

Parameters assessed during the control and intervention phases included: patient characteristics; procedural details; proportion of repeated diagnostic procedures; time intervals (from screening referral to TAVI procedure; time spent in different post-TAVI hospital settings; see below); staff working hours (physicians, nurses, medical technicians, coordinators); patient satisfaction (5-point scale; modified from the principles outlined by Hawthorne et al. [10]); coordinator self-assessment of impact on contacts and hospital logistics relating to TAVI (see below); and clinical outcomes post-procedure and at 30 days.

Assessment of time intervals included various parameters. The mean time from screening referral until the TAVI procedure was assessed for the overall population. Detailed timelines were assessed separately for: (1) patients who were discharged after screening and readmitted later for the TAVI procedure (time from referral until screening, screening duration, and times from screening until admission and admission until TAVI); and (2) patients who were screened and remained in hospital until TAVI (time from referral until admission for screening, time from admission until TAVI). For the in-hospital stay post-TAVI procedure, the length of stays in the intensive care unit (ICU), intermediate care (IMC), and general ward were assessed [11].

The self-assessment for the TAVI coordinators included the effects of their new role on contact with patients and staff (prior to hospitalization, during admission, and post-discharge) and on hospital logistics related to TAVI.

### 2.6. Statistics

This pilot study was intended to determine patient sample sizes for the larger BENCHMARK registry. Baseline characteristics were compared between the cohorts (pre- vs. post-implementation of TAVI coordinator program) using two-tailed t-tests or the Wilcoxon rank sum test for continuous variables and χ2 tests or Fisher’s exact tests for categorical variables (*p* values < 0.05 were regarded as statistically significant). Statistical analysis was performed using SPSS Version 24.0 (IBM Corp., Armonk, NY, USA). 

## 3. Results

At enrollment, 16 patients did not meet the inclusion criterion for a ‘balloon-expandable valve’ because they received a self-expanding valve. The aim of the registry was to examine the possible impact of a TAVI coordinator program on the treatment pathway, rather than the procedure itself, thus, as a result, these patients were included in this pilot study.

### 3.1. Center and Patient Characteristics

Patients were recruited at three German centers including (1) the German Heart Center Munich, which performs >800 aortic valve interventions/year with two specialized teams [12]; (2) the Cardiology Department, Düsseldorf University, which performs 750 TAVIs/year [13]; and (3) the Heart Center Bad Bevensen, a regional hospital in Northern Germany performing about 240 TAVIs/year.

Overall, 84 patients at these centers were documented prior to implementation of the TAVI coordinator program (control phase), and a further 81 patients were documented during the outcomes phase after coordinator implementation (intervention phase). Of these, 34 (pre)/25 (post) were documented in Munich, 26/25 in Düsseldorf, and 25/30 in Bad Bevensen. The overall follow-up completeness was 99% for the TAVI procedure, 99% until hospital discharge, and 97% at 30 days with no marked differences between the centers.

The overall study population (pre- and post-TAVI) had a mean age of 80.2 ± 6.2 years, was 30.9% female, and received a 16.2 ± 10.2% mean logistic EuroSCORE [14,15]. There was a high degree of morbidity in all patients as evidenced by the abundant cardiovascular morbidities and high logistic EuroSCORE. Patient characteristics in the control and implementation phases were comparable with the exception of marginally more patients in the control phase who were symptomatic for AS (88.1% vs. 70.4%; *p* = 0.005) (Table 2). Additionally, fewer patients in this phase had left bundle branch block (LBBB; 3.6% vs. 14.8%; *p* = 0.012). 

### 3.2. Procedural Approach

TAVI coordinators had no responsibility for procedural approaches. The majority of TAVI procedures were performed in a hybrid operating room (83.6%) with patients under conscious sedation (65.5%) (Table 3). Balloon-expandable valves were implanted in most patients (89.7%); a total of 16 patients received self-expanding valves. Procedural times did not significantly differ between the two study phases.

### 3.3. Outcomes: Diagnostic Procedures

**Figure 2:**
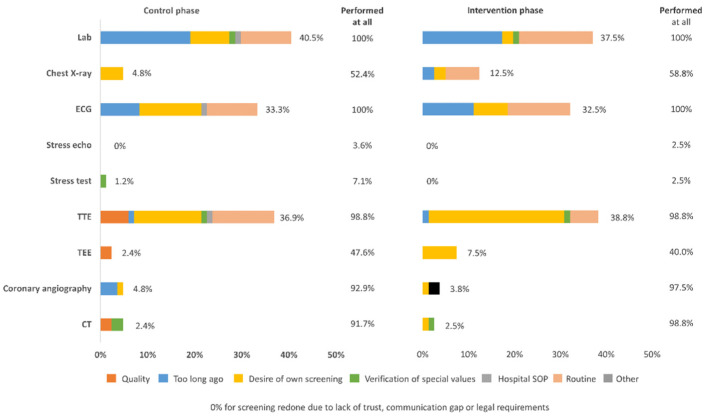
Screening procedures that were repeated, with given reason, for the control vs. intervention phases. CT, computed tomography; ECG, electrocardiogram; SOP, standard operating procedure; TEE, transesophageal echocardiogram; TTE, transthoracic echocardiogram.

Previously performed laboratory values (either externally or in-house) were redone for 39.0% (64/164) of patients overall; transthoracic echocardiograms (TTE) were redone for 38.3% (62/162) of patients, electrocardiograms (ECG) for 32.9% (54/164), and chest X-rays for 15.4% (14/91). Conversely, coronary angiography (7/156; 4.5%) and computed tomography (CT) (4/156; 2.6%) were rarely repeated. The principal reasons for repetition were “test too long ago” (laboratory values, ECG, and coronary angiography), “routine or desire for own screening” (laboratory values, chest X-ray, ECG, TTE, and transesophageal echocardiogram [TEE]), and “verification of specific findings” (stress test, CT) (Figure 2). Standard hospital operating procedures required that some diagnostics, including laboratory values, chest X-ray, ECG, and TTE, be performed in-house irrespective of prior investigations. There were no differences in the rate of repeated diagnostic procedures between the control and intervention phases, although for several parameters this was based on low absolute numbers.

### 3.4. Outcomes: Time Efficiency

Some principal responsibilities for TAVI coordinators with room for improvement were to “coordinate the admission” (two centers), “schedule the diagnostic work-up” (one center), “support early discharge stratification” (i.e., the TAVI coordinator ensures availability of all data to enable early discharge, e.g., arranging an ECG after intervention and ensuring there is adequate patient support post-discharge) (one center), *“arrange internal logistics”* (two centers), and *“schedule post-TAVI diagnostic work-up”* (one center) (Table 1). Addressing these points may result in a streamlined patient pathway or shortened hospitalization.

**Figure 3:**
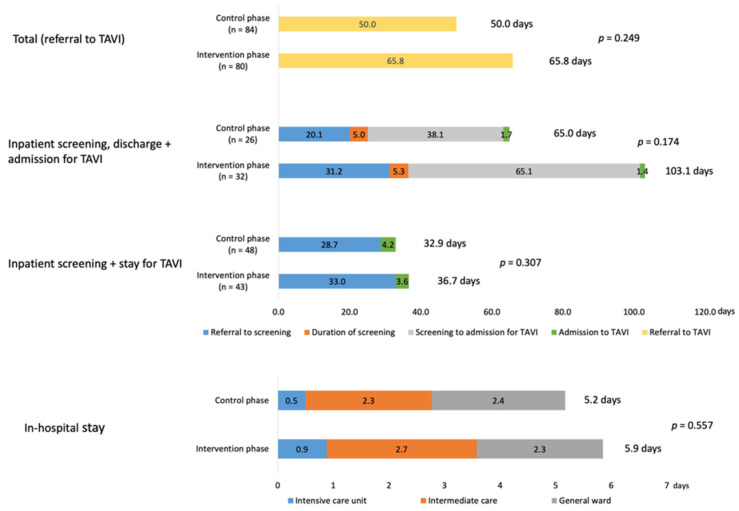
Timeline from referral to TAVI and in-hospital stay for control vs. intervention phases. For the total population, breakdown of the timeline into ‘referral to screening’, ‘duration of screening,’ and ‘time between screening and TAVI’ was not possible because of the need to account for time spent in post-screening discharge for the subgroup of patients who were discharged after screening before later re-admission for the TAVI procedure. TAVI, transcatheter aortic valve implantation.

Interestingly, the mean time between screening referral and the TAVI procedure increased from 50 to 65.8 days in the control vs. the intervention phase (Figure 3). While patients had a longer initial wait after referral to get a combined appointment (28.7/33.0 days vs. 20.1/31.2 days for an isolated screening visit), hospitalization time to perform both screening and TAVI was shorter (4.2/3.6 days vs. 6.7/6.7 days). When screening took place separately, the waiting time between discharge after screening and performance of the TAVI procedure was substantial (38.1 days pre/65.1 days post).

The time between TAVI and discharge from hospital was 5.2 vs. 5.9 days in the control vs. the intervention phase. ICU and IMC unit stays were slightly longer in the intervention phase (Figure 3).

### 3.5. Outcomes: Staff Working Hours

**Figure 4:**
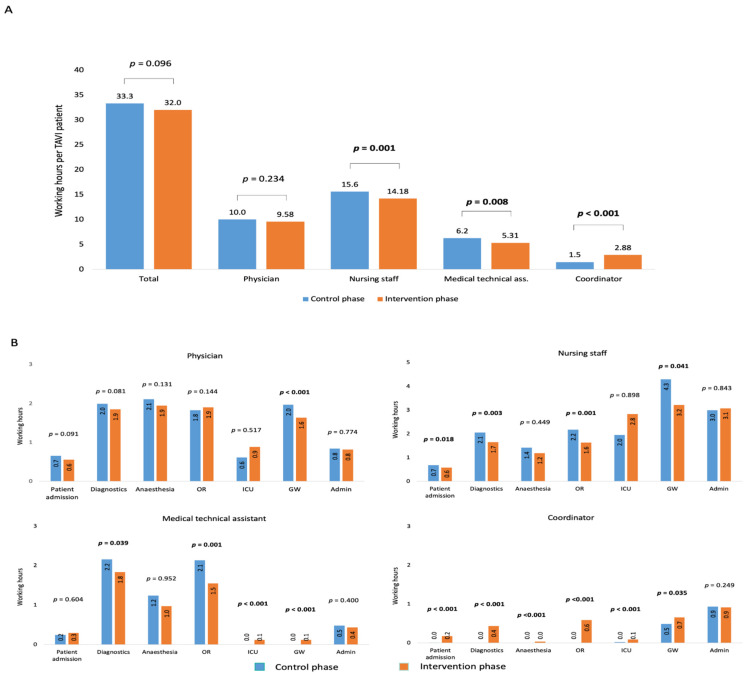
Working hours for physicians, nursing staff, medical technical assistants, and coordinators (**A**) overall and (**B**) differentiated into subcategories for control vs. intervention phases. GW, general ward; ICU, intensive care unit; OR, operating room.

Overall, the implementation of a TAVI coordinator reduced staff working hours (Figure 4), including for nursing staff (15.6 vs. 14.2 h/TAVI patient; *p* = 0.001) and medical technical assistants (6.2 vs. 5.3 h; *p* = 0.008). The main reasons were a significant reduction in time for diagnostics and operating room attendance, but time spent on patient admission (*p* = 0.018) and the general ward (*p* = 0.041) was also reduced for nurses. As expected, the TAVI coordinators’ workloads increased after they assumed the coordinator role (1.5 vs. 2.9 h; *p* < 0.001). 

### 3.6. Outcomes: Patient Satisfaction

**Figure 5:**
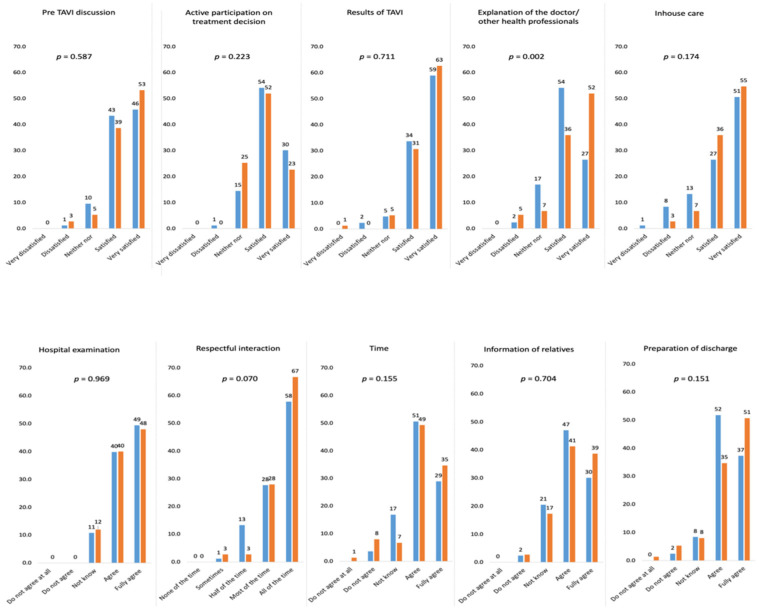
Patient satisfaction subcategories during control vs. intervention phases. Patient satisfaction was assessed using a 5-point scale where 0 = very dissatisfied and 5 = very satisfied (based on principles outlined by Hawthorne et al. [10] and modified to include questions on communication). TAVI, transcatheter aortic valve implantation. Blue-colored bars, control phase; orange-colored bars, intervention phase.

Patients were generally quite satisfied with the treatment pathway (both in the control and intervention phases), with most patients indicating that they were either satisfied (4 out of 5 points) or very satisfied (5/5 points) (Figure 5). Patients generally expressed higher scores during the intervention phase (but with a non-significant statistical test; *p* = 0.408), with the shift being statistically significant for the “explanation of the doctor/other health professionals” (*p* = 0.002). In addition, there were discrete improvements in other areas, e.g., “respectful interaction” (*p* = 0.070). 

### 3.7. Outcomes: Coordinator Self-Assessment 

The self-assessment by TAVI coordinators regarding their impact on contacts and hospital logistics relating to TAVI (Table 4) showed that their pre-hospitalization phone contacts increased (*p* = 0.001), while their personal contacts decreased (*p* = 0.003). Patient contact increased from 69% to 100% during the admission period, and the mean number of contacts during the hospital stay also increased. During in the intervention phase, more supporting material (TAVI information sheets, individualized hospital TAVI sheets, and extended consultations) was provided by the coordinator (all *p* < 0.001). Post-TAVI follow-up contact with the TAVI coordinator was limited, with most contact directed to the referring physician.

The findings from the TAVI coordinators’ self-assessments showed that coordinator implementation increased patient and caregiver expectation setting (*p* < 0.001), as well as support for patient stratification (*p* < 0.001), and improved the coordination of internal logistics/bed occupancy (*p* = 0.008) prior to the TAVI procedure. After the TAVI procedure, the TAVI coordinator increased coordination of patient discharge (*p* < 0.001), patient discharge preparation/management (*p* < 0.001), coordination of internal logistics (*p* = 0.006), coordination of follow-up examinations (*p* = 0.012), and support for early discharge stratifications (*p* = 0.014).

### 3.8. Outcomes: Clinical Outcomes Post-Procedure and at 30 Days

There were no adverse outcomes either post-procedure or at 30 days with the TAVI coordinator program; none of the documented variables differed significantly pre- and post-implementation (Table 5). There was no difference between the groups in the number of correctly positioned single valves (100%), valves with the intended performance (98.8%), and valves considered successfully implanted (98.8%). The frequency of atrioventricular block was nominally lower pre-implementation (3.6% vs. 6.3%), while the number of bleeding complications was lower post-implementation (4.8% vs. 1.3%), but the differences were not statistically significant. 

## 4. Discussion

The COORDINATE study is the pilot study for the larger BENCHMARK registry and was guided by the existing TAVI coordinator role already implemented in Kiel, Germany. COORDINATE involved three distinct, high-volume heart centers in Germany, where the existing TAVI treatment pathways were reviewed, and the impact of implementing a TAVI coordinator to support the clinical team to improve patient management without compromising patient safety was assessed. Overall, 13 responsibilities were suggested for TAVI coordinators. Excluding those responsibilities that had already been implemented, each center selected seven or eight responsibilities that were applicable to their center. The medical center in Kiel with an established TAVI coordinator provided an intensive off-site training period, which was followed by a 1-month implementation period to establish TAVI responsibilities at each center, and then by an intervention phase. A comparison of the new treatment pathway with pre-implementation (control phase) revealed the following:

### 4.1. Diagnostic Procedures

Initial screening procedures, performed externally or in-house, were considered appropriate for coronary angiography and CT. Conversely, routine clinical practice and standard hospital operating procedures required repeat testing in-house for laboratory values, ECG, and TTE. The role of the TAVI coordinator, therefore, did not impact the rate of repeat diagnostic procedures pre- and post-implementation. TAVI coordinator implementation at another German center significantly reduced the repetition of coronary angiography and CT examinations that had been performed at other facilities, however, there was no change in TEE examinations [9]. The differences between COORDINATE and the aforementioned study may, in part, be due to center/region-specific different practices and policies.

### 4.2. Time Efficiency

Implementation of a TAVI coordinator could improve planning and workflow along the TAVI pathway, potentially shortening the treatment pathway [9,18]. The findings from the COORDINATE pilot study showed that TAVI coordinators had little impact on shortening time for diagnostics, admission, and the TAVI procedure itself. For those patients with separate hospitalizations for diagnostics and the TAVI procedure, the delay between screening and TAVI was huge and became even longer after implementation of the TAVI coordinator program. This finding was unexpected, but may be because German DRG regulations stipulate a pre-specified wait time in order to consider diagnosis and intervention as two different hospitalizations. Without this, reimbursement is reduced. Study data has shown that the increased wait time for TAVI is associated with a decrease in functional status (decline in gait speed and increase in frailty) [19]. As a result, increasing the time to TAVI treatment with a TAVI coordinator warrants further investigation to understand why the delay occurs and what can be done to streamline the process to minimize adverse impacts on patient health. Hospitalization with combined diagnostics and treatment are much more time efficient. Another German study showed that the introduction of a TAVI coordinator reduced the time between admission and TAVI from a median 9 (IQR 7–14) to 6 (IQR 3–10) days (*p* = 0.001) [9].

The coordinator had no role in modifying the implant procedure itself, but a number of variables, including the location of the TAVI, the primary valve type employed, and the rate of dilation prior to TAVI, were different in the control and intervention phases. Moreover, the intervention time was prolonged, although this did not reach statistical significance. This is indicative of an inherent and substantial variation in the TAVI procedure itself that cannot be explained by TAVI coordinator implementation. It is likely that any such variations would be less apparent in the larger BENCHMARK study with more centers and patients involved. 

In the COORDINATE pilot, most patients were discharged after 5 days in both the pre- and post-implementation phases. Length of hospital stay is affected by the patient’s risk profile, clinical pathways, and healthcare reimbursement rules [18], but early hospital discharge (e.g., <3 days) is appropriate for select patients [20]. Five days is the minimum stay eligible for full reimbursement in the German healthcare system; therefore, 5 days is probably the shortest time that can be expected in these studies. The length of stay in this pilot study is consistent with that reported for another German study, where the average in-hospital stay for transfemoral TAVI was 10 ± 7 days [11]. Another German study found that a TAVI coordinator program reduced the median post-TAVI hospital stay from 9 (IQR 7–15) to 7 days (IQR 6–11) (*p* = 0.001) [9].

The benefits of introducing a TAVI coordinator were apparent, including providing explanations from the physician and other health officials, respectful interaction between the medical community and the patient, providing information to the patient’s relatives, and preparing the patient for discharge. 

### 4.3. Staff Working Hours

TAVI pathways are complex and involve different medical personnel. Any modifications to the TAVI pathway may affect the staff workload, with associated cost implications [21,22,23]. In this pilot study, implementation of a TAVI coordinator reduced patient-related working hours for nurses (fewer hours on patient admission, diagnostic assessments, and working in the operating room and general ward) and medical assistants (less time on diagnostics and in the operating room). Some of this time was accrued by the TAVI coordinator. As expected, the effects of the TAVI coordinator on physician workload and administrative efforts were limited. To the best of our knowledge, the COORDINATE pilot study is the first study to compare staff working hours pre- and post-implementation of a TAVI coordinator program.

Intriguingly, this observation contradicts the trend in prolonged hospitalization during COORDINATE post-implementation (5.2 vs. 5.9 days in the control and intervention phases, respectively). These differences were not statistically significant; data from the larger BENCHMARK study should be able to provide more information on any potential effects. In an ideal scenario, working hours for medical staff and the duration of hospital stay should be reduced. 

### 4.4. Patient Satisfaction

Patient satisfaction, which is influenced by access to clinicians, treatment timing, the treatment itself, treatment efficacy, availability of relevant information, communication with healthcare staff, and participation in decision making, is an important part of patient-centered care and is used to monitor healthcare provision and develop policies [10]. Support and guidance from healthcare professionals is important for patients awaiting TAVI and can also facilitate the transition to home [24,25].

In COORDINATE, patients were generally satisfied (or very satisfied) with the treatment pathway both before and after the introduction TAVI coordinator. This high baseline value makes the detection of a post-implementation improvement more difficult. Potential improvements were seen for in-house care (*p* = 0.174), respectful interaction (*p* = 0.070), and preparation of discharge (*p* = 0.151), but with only an improvement of explanations provided by health professionals reaching statistical significance. However, there was a trend towards less patient involvement in treatment decisions (*p* = 0.223), but this did not reach statistical significance. Again, the larger dataset for BENCHMARK registry will provide additional valuable information on patient satisfaction.

### 4.5. Coordinator Self-Assessment

Well-coordinated TAVI programs can provide excellent clinical outcomes with reduced hospital stays and with most patients discharged to their home [7,8,9]. Patient safety is paramount, and TAVI coordinators play a key role in optimizing the TAVI pathway by ensuring good communication between all relevant parties, coordinating patient assessments, managing waiting lists, facilitating in-hospital logistics, educating and liaising with patients, and ensuring continuity of care [4,9,18,26,27,28].

This pilot study showed that the TAVI coordinator role resulted in more consistent and intensive contact between the patients and staff, including the provision for supporting materials/information sheets, which may have contributed to the overall increase in patient satisfaction. Furthermore, TAVI coordinators impacted pre-TAVI care, including patient stratification and hospital logistics, as well as post-TAVI care, including the management and logistics of patient discharge. 

### 4.6. Post-Procedure and 30-Day Clinical Outcomes

TAVIs were performed with a high degree of procedural success in both study phases. Introduction of the TAVI coordinator had no adverse effect on the patient’s clinical outcomes post-procedure or after 30 days. The 30-day mortality rate was low (2.6%) and consistent with rates reported for other German and European TAVI centers [9,18,29].

### 4.7. Limitations

Firstly, and as expected, this pilot study had a low number of patients, meaning that potentially clinically relevant differences may not appear to be significant (e.g., the rate of repeated diagnostic procedures pre- and post-implementation of the TAVI coordinator). The larger BENCHMARK study will clarify this data. Secondly, COORDINATE aimed to recruit consecutive patients at each center. However, some patients were omitted due to logistics, which may have resulted in an unknown bias for the population under investigation. Thirdly, the combined implementation and outcome phases may have been too short to have had a substantial impact on the hospital treatment pathway. It may take longer for the new processes to become fully embedded and for significant effects to be seen. Fourthly, the experience gained by the TAVI coordinator and the heart team over the course of the control phase may have contributed to the benefits seen in this pilot study. Finally, patient satisfaction may be dependent on other variables not readily captured in COORDINATE, and the high baseline satisfaction with existing TAVI procedures leaves little room for improvement.

### 4.8. Outlook

The key learning points from COORDINATE were used to design the BENCHMARK registry [30] (ClinicalTrials.gov Identifier: NCT04579445). Briefly, the number of patients for recruitment increased from 165 to 2400 to ensure sufficient power to show statistically significant differences (if present) for the questions under investigation. Secondly, clearer instructions were provided on the potential domains for coordinator involvement. Thirdly, an education phase was introduced for self-assessment of the centers, with education on Quality of Care measures and repeated surveillance of the measures implemented into clinical practice. Finally, recruitment was prolonged to 8 months (vs. 3 months in COORDINATE), and follow-up was extended to 12 months (vs. 1 month in COORDINATE).

## 5. Conclusions

The results of this pilot study, and the experience gained, represent a planning basis for the design of the larger BENCHMARK registry. While the COORDINATE study revealed the beneficial role of a TAVI coordinator, these findings will be verified and validated with the larger study. 

## Figures and Tables

**Table 1:** Center-specific additions to the TAVI coordinator responsibility beyond those already implemented prior to baseline.

	Munich	Düsseldorf	Bad Bevensen
Definition of TAVI coordinator role	X	X	X
Communication with referral physicians			X
Coordination of admission	X		X
Optimizing and standardizing diagnostics	X	X	X
Scheduling of diagnostic workup			X
Frailty screening *			
Patient expectation setting (for the TAVI outcomes)	X	X	X
Support of patient assessment and risk stratification		X	X
Support of Heart Team meetings	X	X	X
Support of early discharge stratification		X	
Arranging internal logistics	X	X	
Scheduling of post-interventional diagnostic work-up	X		
Coordination of follow-up examinations	X		
Total number of selected responsibilities	8/13	7/13	8/13

* There was no specific documentation or training for COORDINATE to document frailty in more detail than is already done in routine clinical practice. X indicates TAVI coordinator responsibilities beyond those already implemented prior to baseline. Empty boxes represent those responsibilities already implemented prior to baseline. TAVI, transcatheter aortic valve implantation.

**Table 2:** Patient characteristics prior to (control phase) and after implementation of the TAVI coordinator (intervention phase).

	Control (n = 84)	Intervention (n = 81)	*p*-Value
Age (years)	80.3 ± 6.7	80.1 ± 5.7	0.867
Gender female, %	31 (36.9)	20 (24.7)	0.090
BMI (kg/m^2^)	26.9 ± 4.6	27.2 ± 5.0	0.604
Symptoms	74 (88.1)	57 (70.4)	0.005
Angina CCS III or IV	9 (10.7)	14 (17.3)	0.223
NYHA class III or IV	63 (75.0)	59 (72.8)	0.752
Syncope	7 (8.3)	3 (3.7)	0.329
Dizziness with exertion	21 (25.0)	18 (22.2)	0.675
Echocardiographic parameters			
Indexed AVA (cm^2^/m^2^)	0.38 ± 0.10	0.38 ± 0.10	0.867
Maximum jet velocity (m/sec)	4.11 ± 0.69	4.00 ± 0.68	0.270
Mean transvalvular PG (mmHg)	42.6 ± 14.3	40.8 ± 13.6	0.419
LVEF < 30%	3 (3.6)	7 (8.6)	0.205
Comorbidities			
Atrial fibrillation	21 (25.0)	15 (18.5)	0.314
Previous MI within 90 days	7 (8.3)	6 (7.4)	0.825
Prior cardiac surgery	15 (17.9)	15 (18.5)	0.912
Peripheral vascular disease	11 (13.3)	5 (6.2)	0.127
Neurologic dysfunction	4 (4.8)	4 (4.9)	0.625
Diabetes mellitus	17 (22.4)	22 (27.2)	0.487
HF within 2 weeks prior TAVI	13 (15.5)	15 (18.5)	0.603
Chronic pulmonary disease	16 (19.8)	9 (11.3)	0.136
Pulmonary HT (>55 mmHg)	8 (9.5)	8 (9.9)	0.939
Renal insufficiency (CrCl ≤ 50 mL/min or dialysis)	16 (19.0)	21 (25.9)	0.290
Further variables			
Frailty (severe) ^a^	3 (3.6)	3 (3.7)	0.999
Impaired mobility	26 (31.0)	16 (19.8)	0.099
Mini Mental State Examination ^b^	27.8 ± 2.0	27.4 ± 1.9	0.456
Logistic EuroSCORE I	16.8 ± 11.0	15.5 ± 9.3	0.415
Social characteristics			
Retired	80 (95.2)	78 (96.3)	0.999
Married	49 (58.3)	48 (59.3)	0.904
Caregiver available	25 (29.8)	33 (40.7)	0.140
Living status			0.521
Living alone	24 (28.6)	17 (21.0)	
With spouse/partner/family	57 (67.9)	62 (76.5)	
Care facility/assisted living	3 (3.6)	2 (2.5)	
Referral physician			0.003
Heart surgeon	1 (1.2)	0 (0)	
Cardiologist	47 (56.0)	63 (77.8)	
General practitioner	29 (34.5)	10 (12.3)	
Other	7 (8.3)	8 (9.9)	
Referral location			0.920
Own hospital	9 (10.7)	9 (11.1)	
Outpatient practice	36 (42.9)	31 (38.3)	
Medical Center (“MVZ”)	2 (2.4)	1 (1.2)	
Other hospital	36 (42.9)	39 (48.1)	
Other location	1 (1.2)	1 (1.2)	

Values are mean ± standard deviation (SD) or n (%). ^a^ Defined as inability to perform two or more activities of daily life (ADL) [16]. ^b^ Mini Mental State Examination (MMSE-2): 0 poor, 30 good [17]. AVA, aortic valve area; BMI, body mass index; CCS, Canadian Cardiovascular Society; CrCl, creatinine clearance; EF, ejection fraction; HF, heart failure; HT, hypertension; LVEF, left ventricular ejection fraction; MI, myocardial infarction; MVZ, Medizinisches Versorgungszentrum; NYHA, New York Heart Association; pts, patients; SD, standard deviation; TAVI, transcatheter aortic valve implantation.

**Table 3:** Procedural characteristics prior to (control phase) and after implementation of the TAVI coordinator (intervention phase).

	Control (n = 84)	Intervention (n = 81)	*p*-Value
Location of TAVI			<0.001
Catheter lab	1 (1.2)	0 (0)	
Hybrid operating room	83 (98.8)	55 (68.8)	
Operation room	0 (0)	25 (31.3)	
Full anesthesia	26 (31.0)	31 (38.8)	0.295
Primary valve type			<0.001
Edwards SAPIEN® 3	59 (70.2)	55 (68.8)	
Edwards SAPIEN® 3 Ultra	10 (11.9)	24 (30.0)	
Edwards CENTERA®	8 (9.5)	0 (0)	
Accurate Symetis	7 (8.3)	0 (0.0)	
NVT Allegra	0 (0.0)	1 (1.2)	
Transfemoral access	84 (100)	79 (98.8)	0.488
Dilation pre TAVI	30 (35.7)	13 (16.3)	0.005
Dilation post TAVI	15 (17.9)	9 (11.3)	0.231
Procedural time			
Induction time ^a^	30.5 (22.0; 53.8)	35.0 (25.0; 53.8)	0.159
Procedural time ^b^	52.0 (44.0; 65.8)	55.0 (44.0; 69.8)	0.382
Intervention time ^c^	96.0 (81.3; 125.8)	105.0 (93.5; 135.0)	0.058
Discharge post TAVI (days) Mean ± SD	5.17 ± 2.51	5.86 ± 4.25	0.571
Median (IQR)	5.0 (4.0; 6.0)	5.0 (4.0; 7.0)	
Discharged within 5 days	55 (65.5)	47 (58.8)	0.375
Discharge direction			0.653
Home	61 (72.6)	54 (67.5)	
Rehabilitation	19 (22.6)	23 (28.8)	
Other hospital	4 (4.8)	3 (3.8)	
Nursing home	0 (0)	0 (0)	

^a^ From start of anesthetic treatment until entering the hybrid operating room; ^b^ from skin incision to closure; ^c^ from start of anesthetic treatment to exit from the operating room. Pts, patients; TAVI, transcatheter aortic valve implantation. Values are mean ± standard deviation (SD), median (interquartile range, IQR) or n (%).

**Table 4:** Level of contact and coordinator self-assessment for pre- and post-TAVI periods, prior to (control phase) and after implementation of the TAVI coordinator (intervention phase).

	Control (n = 84)	Intervention (n = 81)	*p*-value
Contact prior to hospitalization	38 (45.2)	35 (43.2)	0.793
Standard information used			0.965
TAVI information sheet	37 (44.4)	34 (42.0)	
Other	1 (1.2)	1 (1.2)	
Type of contact *			
Phone	6 (7.1)	22 (27.2)	0.001
Personal contact	29 (34.5)	12 (14.8)	0.003
Other	5 (6.0)	7 (8.6)	0.506
Coordinator contact during admission	58 (69.0)	81 (100)	<0.001
Number of contacts during hospital stay	1.98 ± 0.81 2.0 (1.0; 3.0)	2.50 ± 0.57 2.0 (2.0; 3.0)	<0.001
Supporting material			
Pre-TAVI information sheet	58 (69.0)	78 (96.3)	<0.001
Hospital individualized TAVI sheet	0 (0)	26 (32.1)	<0.001
Extended consultation	23 (27.4)	46 (56.8)	<0.001
Other (hospital website, video etc.)	1 (1.2)	0 (0)	0.999
Coordinator contact post-TAVI			
Type of information			
Medical discharge letter	84 (100)	80 (100)	n.a.
Post-TAVI information sheet	33 (39.3)	30 (37.5)	0.814
Phone call	0 (0)	0 (0)	n.a.
Recipient of information/call			
Referring physician	65 (77.4)	71 (88.8)	0.053
General practitioner	59 (70.2)	38 (47.5)	0.003
Rehabilitation/other hospital	32 (38.1)	31 (38.8)	0.931
Other	2 (2.4)	0 (0)	0.497
Self-assessment of support pre-TAVI			
Coordination of admission	25 (29.8)	32 (40.0)	0.169
Optimization/coordination of pre-interven tional diagnostics	19 (22.6)	27 (33.8)	0.113
Patient and caregiver expectation setting	25 (29.8)	51 (63.8)	<0.001
Support of patient stratification (frailty, mental and social status)	24 (28.6)	54 (67.5)	<0.001
Coordinating internal logistics/bed occupancy	24 (28.6)	39 (48.8)	0.008
Support of Heart Team meetings	25 (29.8)	31 (38.8)	0.225
Self-assessment of support post TAVI			
Coordination of discharge	0 (0)	20 (25.0)	<0.001
Support of early discharge stratification	0 (0)	18/19 * (94.7)	0.014
Discharge resource coordination	0 (0)	3 (3.8)	0.114
Scheduling of post-interventional diagnostic workup	2 (2.4)	6 (7.5)	0.160
Patient discharge preparation/management (referral location, logistic considerations, post- hospital care)	0 (0)	26 (32.5)	<0.001
Coordination of internal logistics	0 (0)	7 (8.8)	0.006
Coordination of follow-up examinations	0 (0)	6 (7.5)	0.012

* Information missing for one of the 20 patients with “coordination of discharge”. Pts, patients; TAVI, transcatheter aortic valve implantation.

**Table 5:** Clinical outcomes post-procedure and at 30 days prior to (control phase) and after implementation of the TAVI coordinator (intervention phase).

	Control (n = 84)	Intervention (n = 81)	*p*-value
Procedural outcomes			
Peri-procedural mortality	0 (0)	0 (0)	n.a.
Abort prior insertion of valve/instru ments	0 (0)	1 (1.3)	0.488
Valve positioned, catheter retrieved	84 (100)	80 (100)	n.a.
Complications	2 (2.4)	2 (2.5)	0.999
Atrioventricular block	3 (3.6)	5 (6.3)	0.488
Pacing			0.716
Pacing temporary	2 (2.4)	4 (5.0)	
Pacing permanent	1 (1.2)	1 (1.3)	
Open sternotomy ^a^	0 (0)	1 (1.3)	0.488
Bleeding complication	4 (4.8)	1 (1.3)	0.368
Device malfunction	0 (0)	0 (0)	n.a.
Correct positioning of a single valve	84 (100)	80 (100)	n.a.
Second valve	0 (0)	0 (0)	n.a.
Intended performance ^b^	83 (98.8)	79 (98.8)	0.999
Device success	83 (98.8)	79 (98.8)	0.999
Paravalvular leak mod/severe	0 (0)	1 (1.3)	0.488
Outcomes at 30 days			
Mortality all-cause	0 (0)	2 (2.6)	0.230
Major vascular complication ^a^	0 (0)	1 (1.3)	0.484
Life-threatening bleeding ^a^	0 (0)	1 (1.3)	0.484
Acute kidney injury (II–III)	0 (0)	0 (0)	n.a.
Post-procedural pacemaker implanta tion	6 (7.2)	6 (7.8)	0.892
Stroke	3 (3.6)	1 (1.3)	0.621
Rehospitalization	11 (13.1)	7 (8.9)	0.389
AV related dysfunction ^b^	2 (2.4)	2 (2.6)	0.999
Worse CHF/MV disease ^c^	1 (1.2)	0 (0)	0.999

^a^ Patient with intended transapical TAVI, life-threatening apical bleeding (transfusion of 4 units), hemodynamic instability, conversion to open sternotomy; ^b^ defined as: mean aortic valve pressure gradient ≥20 mmHg, EOA ≤0.9–1.1 cm^2^, and/or Doppler velocity index <0.35 m/s and/or valve insufficiency moderate or severe; ^c^ patient hospitalized due to dyspnea, right pleural drainage, and cardiac decompensation due to concomitant mitral regurgitation. AV, aortic valve; CHF, congestive heart failure; MV, mitral valve; n.a., not applicable; pts, patients.

## Data Availability

Data and other information relating to the TAVI work up are available upon reasonable request from the corresponding author.
